# Differences in Staff Perceptions of Physicians’ and Advanced Practitioners’ Contributions in the Nursing Home

**DOI:** 10.1093/geroni/igaf122.2756

**Published:** 2025-12-31

**Authors:** Brandi Peacock, Kira Ryskina, Tetyana Shippee

**Affiliations:** University of Pennsylvania, Philadelphia, Pennsylvania, United States; University of Pennsylvania, Philadelphia, Pennsylvania, United States; University of Minnesota, Minneapolis, Minnesota, United States

## Abstract

For the 1.4 million individuals who receive post-acute care in US nursing homes (NH) annually, timely access to physician or advanced practitioner (PAP) services is federally mandated. However, patient experience indicates a highly variable approach to PAP NH practice between facilities. This study aimed to qualitatively explore the perceptions of PAP contributions to NH care quality by triangulating interview data from key personnel. Purposive sampling was used to recruit 65 participants from a representative sample of 47 NHs in February - November 2023. Eligible participants included all clinical and non-clinical staff who spoke English. We used a grounded theory approach to analyze data collected from one-time semi-structured telephone interviews, divided into two groups: (1) 29 participants from 11 facilities with more than one participant; and (2) 36 solo participants from their facility. Participants included administrators (35%), PAP (17%), nurses (27%), and other staff (21%, e.g. social workers). Among participants from the same facility, 64% disagreed about the frequency of PAP visits. Two major themes emerged. First, administrators were primarily concerned with the minimum necessary presence of PAP at the facilities, whereas nurses perceived general accessibility of PAP as more important. Second, administrators, nurses, and other NH staff perceived limited roles for PAP in care coordination, whereas PAP perceived themselves to take the lead on care coordination for their patients. These findings highlight the need for transparency of expectations of PAP on-site presence and accessibility and for role clarity in care coordination to enhance the quality of PAP contributions in NHs.

